# Impact of high malaria incidence in seasonal migrant and permanent adult male laborers in mechanized agricultural farms in Metema – Humera lowlands on malaria elimination program in Ethiopia

**DOI:** 10.1186/s12889-020-8415-4

**Published:** 2020-03-12

**Authors:** Wossenseged Lemma

**Affiliations:** 1 Department of Medical Parasitology, School of Biomedical and Laboratory Sciences, College of Medicine and Health Sciences, University of Gondar, P. o. Box - 196, Gondar, Ethiopia; 2 Tropical Infectious Diseases Research Center (TIDR), University of Gondar, Gondar, Ethiopia

**Keywords:** Migrant and permanent laborers, Agriculture fields, Confirmed malaria cases, Malaria elimination program, Metema – Humira lowlands, Northwest Ethiopia

## Abstract

**Background:**

Seasonal migrant and permanent laborers who are working in big mechanized agricultural farms in Metema – Humera lowlands are not included in Ethiopia Malaria Elimination Program. The aim of this research was to show the high confirmed and treated malaria cases in these laborers.

**Methods:**

A retrospective analysis of the confirmed and treated malaria cases in all the districts of West, Central and North Gondar Zones, using Weekly Public Health Emergency Management (PHEM) reports, was conducted to show a complete picture of the malaria incidences in the areas.

**Result:**

A total of 3,485,646 confirmed malaria cases were treated in Amhara region during 2013 to 2017. Of the total malaria cases in the Amhara region during these period, 1, 286, 848 cases or 37.2% were originated from West, Central and North Gondar Zones. But these 3 Zones contribute only 17% of Amhara region population. Of all the confirmed malaria cases reported in the 3 Zones, 41.7% (536,749/1286, 848) was reported from the three lowland districts (Metema, West Armachiho and Quara) of the West Gondar Zone during the same study period. But, the West Gondar Zone has only around 10% of the population in these three zones. The highest annual parasite incidence for malaria was found in West Armachiho district. Majority of above 14 years malaria cases in West Gondar zone were found from laborers.

**Conclusion:**

Migrant and permanent laborers working in mechanized agricultural fields in Metema – Humera lowlands are highly exposed to malaria and immediate interventions are required.

## Background

Malaria control by universal coverage of long-lasting insecticidal nets (LLINs) and indoor residual spraying (IRS) in conjugation with effective diagnosis and case managements in endemic areas of the world averted more than 670 million malaria cases since 2000 [1]. Following the first draft national strategic plane to prevent, control and eliminate malaria in Ethiopia during 2011–2015 [2], the second Presidents’ Malaria Initiatives (PMI) Strategy for 2015–2020 [3] was launched to achieve similar goals planned previously: reducing malaria related death to near zero or 1 case /100,000 risk population, elimination of malaria from selected low transmission areas and reduction of malaria by 75% from baseline of 2013 during 2020. But, seasonal migrant adult male agricultural laborers in malaria endemic Metema - Humera and Benshangul lowlands were not included in these planes [3–5]. Seasonal labor migrations from highlands, mainly from Amhara and Tigray regions, are fueled by shortage of farmland and lack of alternative source of income locally. Travel to the lowlands has become a stable livelihood basis both for highlanders and mechanized farm owners in the lowlands [6]. North, Central and West Gondar Zones have around 3 million people (3.6% of total Ethiopian population) but registered 20.9% of all the confirmed malaria cases reported in Ethiopia in 2013 which could be related to the high malaria cases of seasonal migrant and permanent laborers in big mechanized farms of highly malaria endemic Metema – Humera lowlands [7]. The West Armachiho and Metema lowlands were reported among the top 5 districts for malaria incidences from the 166 districts in the Amhara region [8]. The Ethiopian Malaria Elimination Program utilizes households for application of IRS and provision of LLINs [3–5]. But, the migrant laborers have no address to get the privilege of this program. They move from one farm to the next depending on the demand for wedding or harvest of sesame, sorghum and cotton. In the farm either they sleep in an open thatch roofed shelter or open air where they were performing weeding or harvesting. Seasonal laborers working in the farms are an easy prey for mosquito bites and high incidence of malaria. Very few laborers in mechanized farm could afford to buy LLIN from the black market [7]. To alleviate the malaria cases of seasonal laborers in different farms, the West Gondar Zone Health Bureau has recently started seasonal mobile on venue malaria screening and treatment of laborers in addition to in some big mechanized farms during August – November weeding and harvest seasons. A significantly high number of laborers are also seen treated in the government and private treatment centers including some mechanized farms equipped with clinics for the laborers. But, there is no an easy access for treatment in remote agriculture fields and when they are crossing Ethiopian border to work in Gadarif states. Malaria transmission in the seasonal laborers could also be enhanced due to outdoor activities of the laborers especially during harvest time of sesame which is performed at night [9]. President Malaria Initiative (PMI) working in Malaria Elimination Program of Ethiopia has reported the existence of an ongoing research to better understand malaria transmission among migrant workers [5]. So far there is no effective vector control methods applied in mechanized organic agriculture fields appropriate for preventing malaria transmission by affecting the longevity of the vector except using LLINs. The aim of this research was to show the high incidence of malaria cases in laborers working in mechanized agricultural farms in Metema – Humera lowlands for possible awareness creation of stakeholders for possible immediate measures to prevent morbidity and mortality in these mobile laborers in addition to the re-introduction of malaria into the highlands areas by returnees from the lowlands.

## Methods

### Study areas and malaria ecologies

The former North (Semen) Gondar Zone has been now divided into West, Central and North Gondar Zones in Amhara Regional States. The West, Central and North Gondar Zones have a total population of 3,036,961 (1,539,341 Male; 1,497,620 Female) with 85.3% living in rural and 14.7% in urban areas [10]. The West Gondar Zone has a total population of 243, 797 in the Metema, Quara and West Armachiho districts. The Central Gondar Zone includes the Gondar town and the surrounding areas while the new Northern Gondar Zone (high land areas) is found mostly in the northern colder areas which include the Dabat and Debark towns in addition to the Semen mountain national park.

**Fig. 1:**
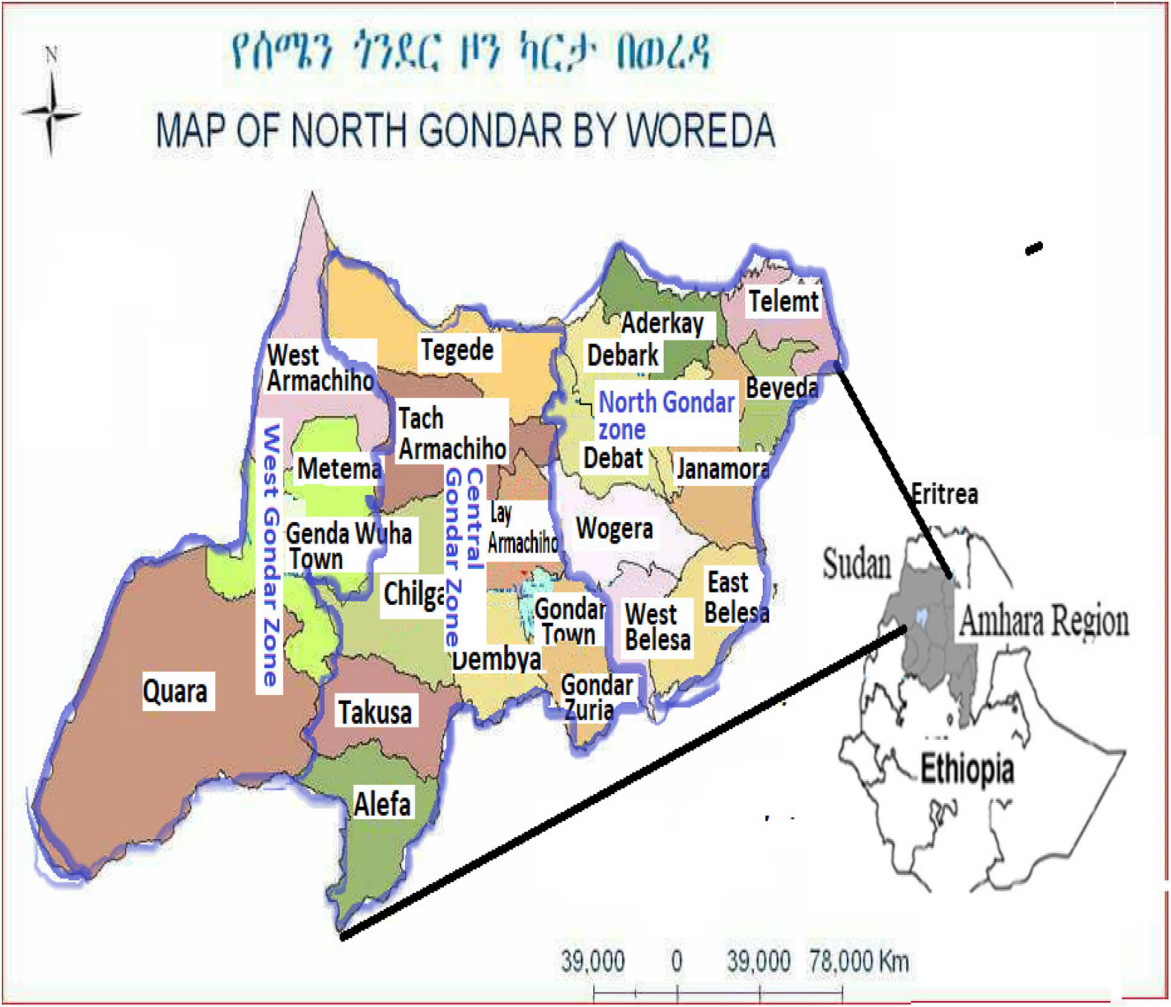
Map of Amhara region in Ethiopia. The map shows West, Central and North Gondar Zones and their districts where seasonal laborers working in the lowlands during agricultural rainy seasons. (Source: Amhara Regional state (http://www.amharabofed.gov.et/). The author acknowledges the source of the figure for use to indicate the study sites of this research. The Image may be subject to copyright

The West Armachiho district is one of the districts in the West Gondar Zone with a total population of 34, 473 (18,919 men and 15,554 women) and is surrounded by Metema (south), Sudan (west), Tigray Region (north) and Central Gondar (Tsegede) (east). The Metema district has a total of 115, 285 population (61,387 male and 53,898 female) who live in urban (32,286) and rural (82,999) areas. It is surrounded by Quara district on the South, West Armachiho on the north, and Central Gondar on the East and Sudan on the west. Quara district has a total population of 96, 782 (51, 415 male; 45, 367 female) in urban (5, 737) and rural (48, 560) areas. It is surrounded by Sudan on west, Metema on the north, Benishabgul-Gumuz on the South and the Central Gondar on the east direction (Fig. 1).

The malaria ecologies in the West, Central and North Gondar Zones can be classified as lowland (< 1500 m asl), lower highland (1500–2000 m asl) and upper highland malaria (> 2000 m asl). Metema (including Genda Wuha town), West Armachiho and Quara lowland districts are found in West Gondar Zone while Tach Armachiho, Chilga and Tegede lowland districts are found in the Central Gondar Zone. The lower highland malaria ecology is found in the districts surrounding the Lake Tana (1800–2000 m asl) which include Dembia, Alefa, Takusa, Gondar Zuraia and Belessa districts. Gondar town, Lay Armachiho, Dabat district, Dabat town, Debark town, Wegera district, AdiArkay, Beyeda, Tselemt and Janamora are the districts and the towns located above 2000 masl and are grouped in the upper highland malaria ecology.

### Malaria elimination program and public health emergency management (PHEM) malaria reporting system

Malaria elimination program in West, Central and North Gondar Zones is part of the Ethiopian Malaria Elimination Program. Ethiopian Malaria Elimination program use households for application of IRS, distribution of LLINs and community based public health education. The smallest administration unit in Ethiopia is called Kebele both in urban and rural areas. The households are placed far apart in the rural kebeles while clustered together in the urbans or towns. Around five hundred households in the same geographical location form one Kebele. In one kebele, there is one health post to provide health service for the residences. A typical health post has 2 health extension workers with two year training after graduating from a high school. The health extension workers are hired by Ethiopian Federal Ministry of Health to provide curative health care services including for malaria in addition to public health awareness creation. More than 80% of malaria cases in the adults and above 5 years old children were also screened using malaria RDTs and treated as outpatients by Health extension workers [5]. The distribution of LLINs to households (1 LLIN per 2 individuals) every 3 years is conducted according to the Health Extension Workers information. The overall IRS is supervised by the Health Extension Workers.

Public Health Emergency Management (PHEM) is a paper-based data collection system developed in 2009 to collect weekly disease reports from health posts, health centers, and hospitals, mostly related to malaria morbidity. Health extension workers in health posts and Health clinics (centers) are responsible to report the malaria cases. The reporting system includes confirmed malaria cases by species in age group less than 5 years, between 5 and 14 years and above 14 years old for both the outpatients and inpatients. The head of a Health Clinic is also the head of the health posts and co-ordinates all the activities of the health posts including the malaria reporting system to the district Health Bureau malaria expert. In the district health bureau, confirmed malaria case reports from all health posts, health clinics and usually from a district hospital are compiled before submitted to Zone Health Bureau. Always a copy of signed and stamped weekly confirmed malaria PHEM reports are attached to health clinic archives after sending to districts and so on.

### Seasonal migrant laborers and malaria cases managements

Annually, mechanized agriculture for cultivation of sesame, cotton and sorghum for commercial purpose in Metema - Humira lowlands, attracts laborers mainly from highland areas of Amhara and Tigray regional states of Ethiopia. Every year, over 200, 000 seasonal migrant laborers come to Kafta-Humera lowlands in Tigray regional state and around 200,000–300,000 to West Gondar Zone to participate in weeding and harvest of these crops before returning home, mostly in three to six months [6, 11, 12]. About 80,000 migrant laborers also cross Ethiopian border from northwest Ethiopia to find work in the neighboring Gedaref State in Sudan where laborers are highly demanded [13]. Almost all permanent residents in the Metema – Humera lowlands live in Gendawuha, Metema Yohannis, Quara, Midre-Genet (Abdurafi), Abrajira, Mykadra, Humera, Dansha, Beaker, Adebay and Rawiyan towns and rural villages in Amhara and Tigray regions. The migrant laborers freely move between the big farms in the lowlands in both Amhara and Tigray region seeking for work mainly during May – November agricultural season. The towns are the main centers for laborers to station temporarily before hired to work in mechanized agricultural fields. These towns are also the main centers for laborers for spending dry non-agricultural season for those who failed to return to their families in the highlands for different reasons. That means they become permanent resident laborers in the lowlands. After November and December harvest season of sesame and sorghum in the Metema – Humera lowlands, some laborers often cross Sudan border to be hired in sorghum harvest until the next agriculture activities in the Metema - Humera lowlands which start mostly in May. There are also laborers who work in Sudan even during rainy July and August sesame weeding time.

The Humera Kahsay Zonal Hospital in the Tigray Region and the Metema and the Abrajira District Hospitats in the Amhara Region and several health clinics and health posts in both regions are the places where these mobile migrant laborers and permanent laborers get malaria treatment. There are also private clinics where laborers get malaria treatments with affordable price in both regions. Amhara Region Health Bureau (West Gondar Zone) initiated seasonal (August–November) screening and treating malaria cases in mobile treatment venues and selected agricultural camping sites, primarily to serve laborers.

Each mechanized agricultural farm has usually one camping site where farm managers, guards and cooks live. In the camp or near the camp, there could be open thatch roofed houses that will serve as sleeping placed for a group of laborers. Some more such thatch roofed shelters for laborers may be found in different part of the farm. But, construction of such shelters in the different part of the farm is rare and migrant laborers usually cook and sleep under trees in the farm [9]. In Metema - Humera lowlands, only households in the towns and rural villages are included in the Malaria Elimination Program. Houses in agricultural camps and thatch roofed shelters for laborers are not sprayed with insecticides. LLINs are not distributed for seasonal laborers. That means, around 500, 000 migrant laborers annually visiting Metema – Humera lowlands are not considered in Ethiopia Malaria Elimination Program. These laborers working in the farm are an easy prey for mosquito bites and commonly infected with malaria.

### Study design

Retrospective analysis of confirmed malaria cases were conducted using Electronic copies of weekly district malaria PHEM reports from all districts in the Amhara Region to evaluate the burden of malaria in West, Central and North Gondar Zones. The statuses of malaria endemicity in different districts and the three malaria ecologies in the three zones (based on altitudinal differences) were analyzed using the appropriate statistics for under 5 years, 5–14 years and above 14 years age groups. PHEM reports in the 20 districts and 3 capital towns in West, Central and North Gondar Zones have also indicated high malaria incidences in the 3 lowland districts (West Armachiho, Metema and Quara) in West Gondar Zone. Of the three districts in West Gondar Zone, West Armachiho district was systematically selected to demonstrate high malaria incidence in laborers working in the agricultural farms. Almost all areas of West Armachiho district is used for mechanized agriculture compared to Quara district where significantly high proportion of the area is occupied by Alatish park bordering with Dendir Park in Sudan. It is also a district with very low number resident populations in towns and rural villages. In the West Armachiho districts, there are 1 district hospital (Abrajira hospital), 2 health clinics (Abdurafi and Abrajira Health clinics) and 9 health posts (Karhomer, Grar Wuha, Mogese, Durmaga, Gobla, Zemene Merik, Torka, Enzlish and Meharish health posts). Data about the weekly confirmed malaria PHEM reports of these health service facilities and from temporary seasonal mobile and agricultural camps treatment centers and a district hospital were manually entered into spss 20 to analyze the trend of malaria in migrant laborers.

### Data collection

Excel documents obtained from Amhara health Bureau and former North Gondar Zone has RDT or microscopy confirmed malaria cases which were identified by species for less than 5 years, 5–14 years and above 15 years age groups for every WHO malaria epidemic weeks for all districts. *Plasmodium falciparium* mono-infection and *Plasmodium falciparium* and *Plasmodium vivax* double infections are considered as *P. falciparium* infection as both infections are treated by Arthmecine based combined therapy (ACT). Only *Plasmodium vivax* mono-infections are considered as *P. vivax*. The excel documents were used to generate spss data for statistical analysis to show differences in confirmed malaria cases in different districts during the different epidemic weeks. The data was also used to compare confirmed and treated malaria cases in under 5 years, 5–14 years and above 14 years age groups. The prevalence of confirmed malaria in under 5 years children was used as an indicator for the endemicity of the districts for malaria. Weekly confirmed malaria PHEM paper reports for confirmed malaria cases from Grar Wuha, Mogese, Durmaga, Gobla, Zemene Merik, Torka, Enzlish and Meharish health posts, Abdurafi and Abrahajira Health Clinics, mobile and agricultural camps seasonal treatment centers and Abrajira Hospita were used to analyze confirmed and treated malaria cases in the West Armachiho district. Retrospective analysis of malaria cases treated in West Armachiho were mainly used to demonstrate high incidence of malaria in laborers working in agriculture fields.

### Quality control

In an effort to reduce malaria related death to near zero, health personals in the districts of West Gondar zone facilitate the malaria screening and treatment process seriously. Malaria screening and treatment is conducted for free in all government health facilities. Although some weekly reports were missing in the remote health posts, enough data reports were analyzed from different treatment centers in West Armachiho district. The weekly malaria PHEM paper reporting in Ethiopia made all district, Zone, Regional and National Health Bureau to have weekly malaria epidemiological data. Copies of the malaria PHEM reports are signed and stamped before attached as files for retrospective uses in District, Zone and Regional Bureau. The organized reporting system and the involvement of stakeholders at different level in Ethiopian Malaria Elimination Program made the malaria cases paper reports excellent in different health bureau in Ethiopia.

### Statistical analysis

Descriptive statistics was used to show and compare the total malaria confirmed cases including for under 5 years and 5–14 years age groups in different districts in West, Central and North Gondar Zones. Descriptive statistics was also used to show confirmed malaria cases treated in different health facilities in West Armachiho districts during the study period**.** Kruskal-Wallis Analysis of Variance (ANOVA), Median overall tests and Mann Whitney U-tests were used to show statistically significant difference for weekly malaria confirmed cases in different districts and different altitude malaria ecologies. P-vales below 0.05 were considered as statistically significantly different.

## Result

### Confirmed malaria cases in west, central and North Gondar zones

A total of 3,485,646 malaria cases were treated in all Zones (districts) in Amhara region during 2013–2017. During the same period, a total of 1286, 848 confirmed malaria cases were reported in West, Central and North Gondar Zones which accounted for 37.2% of all the malaria cases in Amhara region (Table 1). But, the three Zones have only 17% of the Amhara region population. Of the West, Central and North Gondar Zones, 41.7% (536,522/1286, 848) of confirmed malaria cases were reported from the West Gondar Zone during the five years. But, West Gondar Zone has only around 10% of the population in these three zones (Table 1).

**Table 1:** Median, mean, standard deviation and sum of weekly PHEM malaria case reports for districts in West, Central and North Gondar Zones during 2013–2017 and three different malaria ecologies

Malaria Ecologies	District	Median (Mean) ± SD (Sum)			*p*-values	
		Under 5 years	5–14 years	All age group	P-value	P-value
**Lowlands (< 1500 m)**	**Metema**	**71 (78 ± 41.9 (23323)**	**97 (1337 ± 106.7)(39,714)**	**517 (770 ± 611.5)(228,887)**	0.000	0.000
	**pop.:115285**					
	**Mirab Armachiho**	**17 (20.99 ± 15.8)(6214)**	**45 (59.4 ± 50.8 (17591)**	**256.5 (431.04 ± 432.1 (127589)**		
	**Pop.:34473**					
	**Quara**	**27 (34.3 ± 27.9)(19,189)**	**76 (83 ± 47.1)(24,661)**	**374 (424.9 ± 262.9 (126213)**		
	**Pop.:96782**					
	**Tegede**	**33.5 (38.2 ± 22.8)(11,303)**	**67 (80.5 ± 50.8)(23,841)**	**256.50 (345.67 ± 254.5)(102,318)**		
	**Pop.:71436**					
	**Tach Armachiho**	**50 (54.3 ± 32.5)(16,066)**	**85 (97.9 ± 64.7)(28,984)**	**275 (330.1 ± 229.4)(977.10)**		
	**Pop.:92492**					
	**Chilga**	**20 (22.8 ± 15.4)(6804)**	**34 (41.5 ± 28.4)(12,378)**	**187.5 (236 ± 149.9)**		
	**Pop.: 229288**					
	**Genda Wuha**	**22 (26.7 ± 18.1)(7939)**	**24 (29.2 ± 22 (8674)**	**146 (182 ± 110.9)(54,060)**		
Lower highlands (1500-2000 m)	**Dembia**	**24 (29 ± 28.5)(8614)**	**55 (68.6 ± 54.7)(20,376)**	**292 (334.7 ± 221.1)(99,407)**	0.000	
	**Pop.**: **280582**					
	**Takusa**	**7 (8.9 ± 6.7)(2639)**	**14 (16.8 ± 12.04 (4974)**	**83 (93.03 ± 50.7)(27,538)**		
	**pop.: 133391**					
	**Alefa**	**7 (8.41 ± 5.8)(2506)**	**17 (19.7 ± 12.9 (5880)**	**142.5 (166.5 ± 84.4)(49,623)**		
	**Pop.:176313**					
	**Mirab Belessa**	**7 (9.9 ± 10.9)(2932)**	**14 (19.8 ± 18.2 (5884)**	**76 (94.1 ± 62.7)(27,938)**		
	**Pop.146820**					
	**Misrak Belesa**	**13 (19.41 ± 18.5)(5766)**	**23 (38.8 ± 44.3)**	**83 (128.7 ± 135.2)(38,225)**		
	**pop.:101541**					
	**Gondar Zuria**	**10 (21.5 ± 30.3 (6375)**	**19 (37.9 ± 52.7)(11,253)**	**137 (184.1 ± 166.8 (54669)**		
	**Pop.196229**					
**Upper highlands (> 2000 m)**	**AdiArkay**	**4(5.2 ± 5.5)(1535)**	**9 (11.3 ± 10.1)(3367)**	**49 (57.3 ± 38.5)(17,064)**	0.000	
	**Beyeda**	**0(0.02 ± 0.2)(7)**	**0 (0.2 ± 0.6)(56)**	**6 (7.5 ± 6.8)(2240)**		
	**Pop.:100065**					
	**Dabat**	**4(6.1 ± 7.9**)(**1828)**	**14 (19.6 ± 21.6)(5853)**	**86 (94.8 ± 68.2)(28,257)**		
	**Pop.:150792**					
	**Wegera**	**2 (2.6 ± 32)(761)**	**6 (8.1 ± 9.9)(2413)**	**56 (67.5 ± 48.5 (20038)**		
	**Pop.:230438**					
	**Debark Town**	**0 (0.1 ± 3**)(**18)**	**0 (0.2 ± 1.4 (63)**	**7 (8.9 ± 7.1)(2638)**		
	**pop.:166198**					
	**Gondar town**	**7.8 (4 ± 6.4)(1896)**	**7 (11.8 ± 13.8)(3501)**	**94 (110.3 ± 64.8)(32,760)**		
	**Pop.:225125**					
	**Janamora**	**0 (0.7 ± 5.4)(208)**	**3 (3.7 ± 4.1)(1084)**	**29 (36.97 ± 28.9)(10,979)**		
	**Pop.: 173106**					
	**Lay Armachiho**	**4 (6.2 ± 69**)(**1830)**	**14 (17.4 ± 16.7)(5168)**	**94 ± 135.6 ± 129.4 (40258)**		
	**Pop.:163320**					
	**Tselemt**	**0 (2 ± 5.1)(593)**	**1 (5.6 ± 11.97)(1645)**	**22 (31.6 ± 427)(9341)**		
	**Wogera**	**1(2.3 ± 28**)(**669)**	**7 (8.9 ± 8)(2637)**	**51.50 (62.35 ± 43.04)(18,455)**		
	**Pop.: 230438**					
	**Total**	**7 (17.6 ± 26.3 (120109)**	**16 (35.4 ± 51.9 (241609)**	**98 (188.4 ± 267.2)(1,286,848)**		

**Fig. 2:**
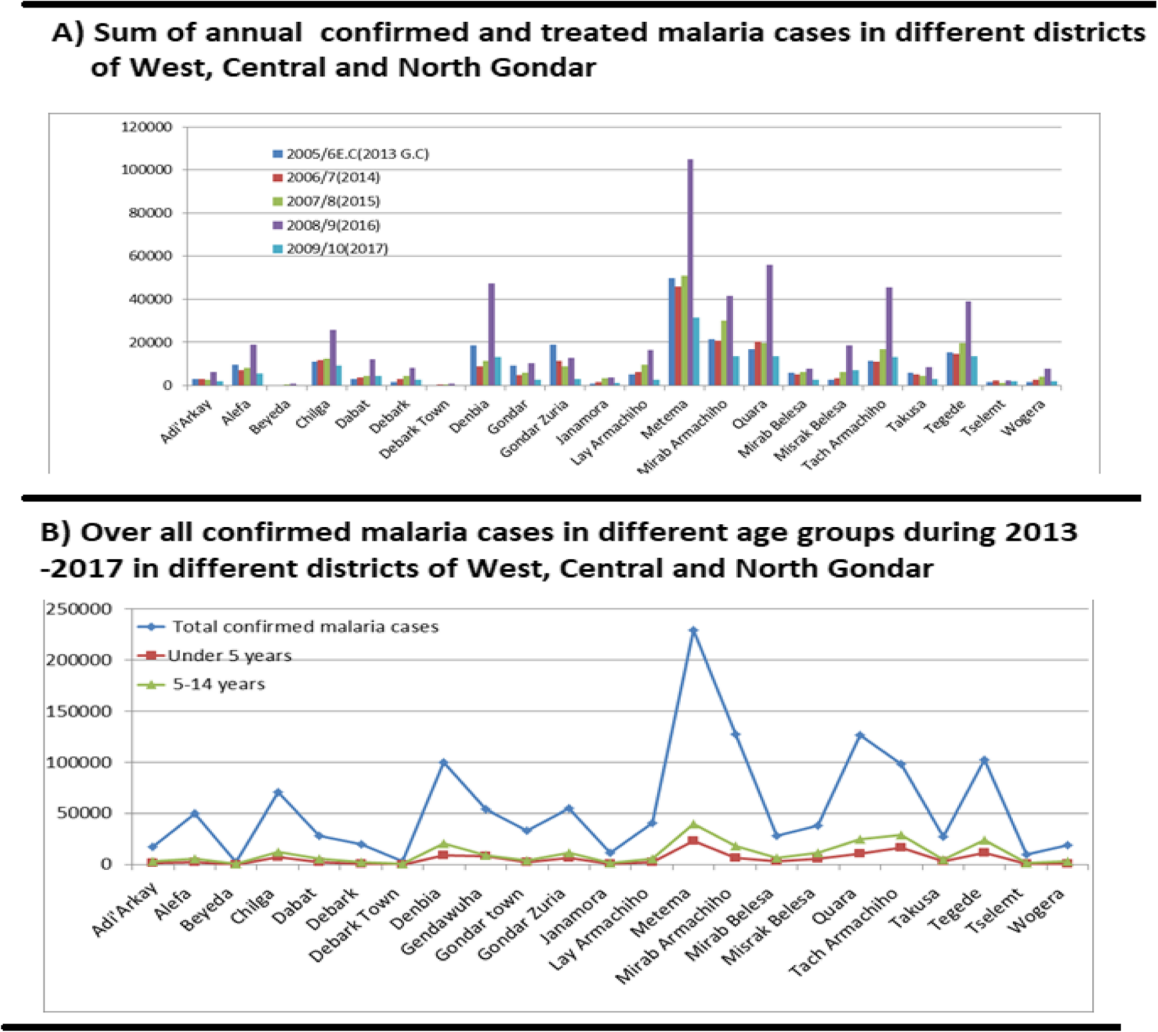
Comparisons of sum of Confirmed malaria cases in different in different districts in West, Central and North Gondar zones for different years (A) and sum of confirmed malaria in different age groups during 2013 to 2017 (B)

On average, around 107, 304 confirmed malaria cases were reported in this West lowland zone annually where more malaria cases were detected from laborers working in agriculture fields. Including the estimated 200, 000 seasonal labors visiting the areas, a total of around 446,540 people are estimated to be exposed to malaria in West Gondar Zone. Parasite incidence rate in West Gondar Zone were calculated as 357 malaria cases/1000 population for year 2013, 287 malaria cases/1000 population for year 2014, 225.7 malaria cases/1000 population for year 2015, 453.6 malaria cases/1000 population for year 2016 and 130.6 malaria cases/1000 population for year 2017. When total annual confirmed malaria cases analyzed for the different districts, the highest (228,887 cases) during 2013–2017 were reported from Metema district with total 115, 285permanent resident population (Table 1). In all districts the number of confirmed malaria cases were statistical significantly different (*p* = 0.000) for years from 2013 to 2017 with the highest malaria cases in all districts in 2016 (Fig. 2a). Permanent residents were living in insecticide sprayed households and using LLINs. They were rarely seen treated for malaria in the health facilities compared to the laborers. The lower malaria occurrence in permanent residents in low land districts could be observed from the very low proportion of malaria occurrence in under 5 years infants (maximum: around 10%) and 5–14 years age groups (maximum: around 20%) (Table 1; Fig. 2b).

Kruskal-Wallis and Median overall tests for different districts and the three different malaria ecologies have showed statistically significantly different malaria incidences (*p* = 0.000) for different districts and malaria ecologies. But during pairwise comparisons between two districts, only few districts in North most colder region have showed for lack of differences (*P* > 0.1): Beyeda–Debark Town (*p* = 0.7), Tselent-Janamora (*p* = 0.105), aderkay-wogera (*p* = 0.4) and Aderkay-Dabat town (*p* = 0.136).

**Fig. 3:**
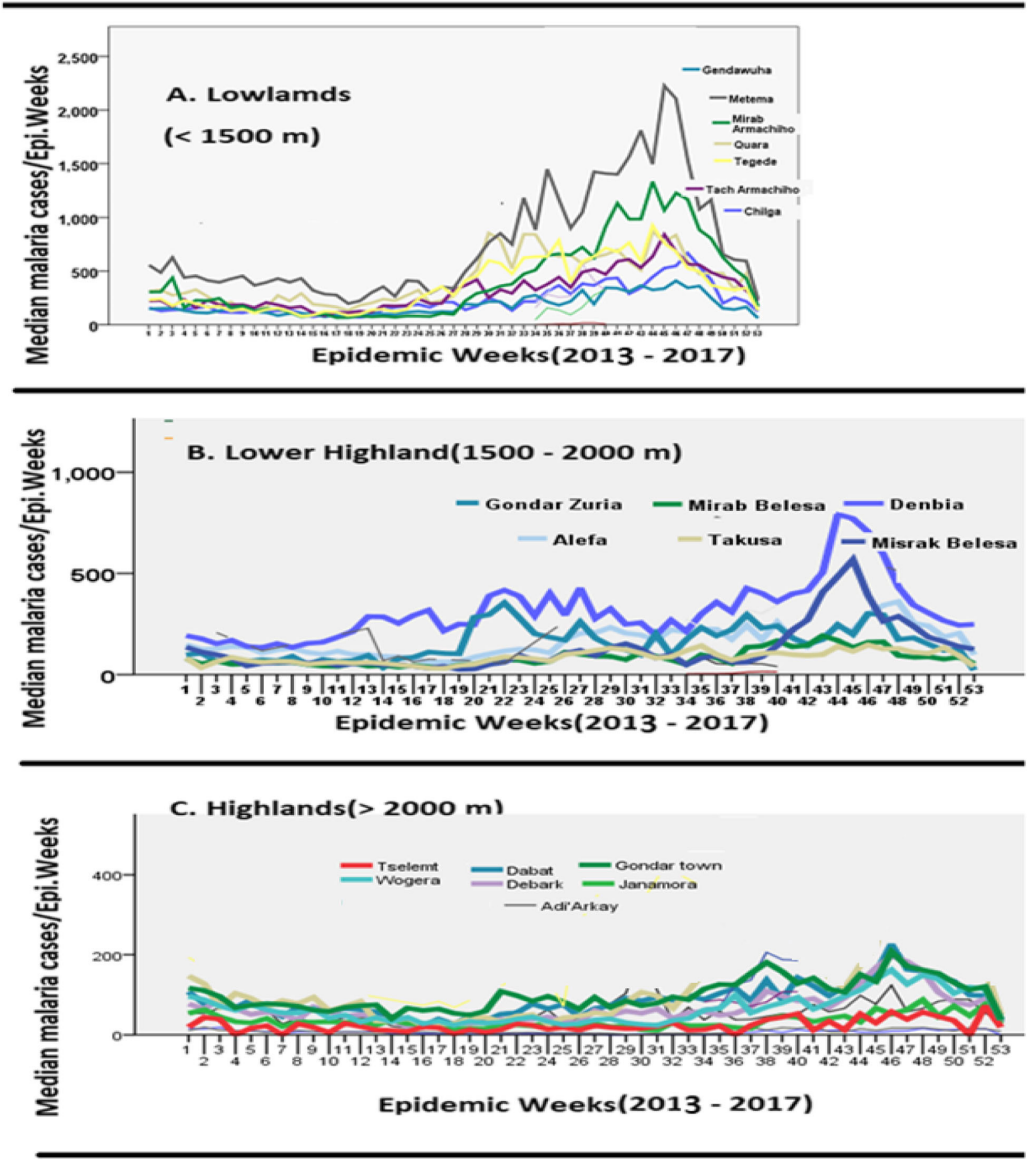
Malaria seasonality during different WHO epidemic weeks during 2013–2017 in Lowland, lower highland and upper highland areas of West, Central and North Gondar Zones

Peak malaria season for lowlands, lower highland and upper high land districts were observed in October (Fig. 3). The highest median peak weekly malaria treated cases (2200 median cases/week) was found in Metema in lowland districts while the highest peak malaria cases (around 800 median cases/week) for the lower highland was found in Dembia district. The highest peak around 200 cases/week for upper highland malaria areas was found in Gondar Town (Fig. 3). Stable low transmission of malaria was found in lowlands, lower highlands and upper highlands throughout the dry season which might be related with the presence of rivers. Minor malaria transmission peak observed only in lower highland areas (Fig. 3).

### Confirmed malaria cases in west Armachiho district

A total of 92, 271 confirmed and treated malaria cases in Health posts, Health clinics, seasonal temporary treatment centers and the district Hospital were documented in weekly malaria PHEM reporting papers for the permanent residents and laborers in West Armachiho District during 2014/15–2017/18. A total of 13, 043(90.6% PF and 9.4% PV) confirmed and treated malaria cases were documented for the temporary seasonal (August – November) mobile and agricultural camps treatment centers during 2015–2017. Almost all (99%) treated in mobile and agriculture camps were male adults aged from 15 to 80 years (Table 2). The total number of 44,637 confirmed malaria cases (68.7% PF and 31.3% PV) were treated in the 9 Health Posts of West Armachiho districts during 2014/15–2017/18. Of all treated cases in the different health Posts of West Armachiho District, 73.8% were males and 26.2% were females. On the other hand, from the total 34,591 confirmed malaria cases (67.1% PF and 32.9% PV) were treated in the Abdurafi and Abrajira health Clinic during 2014/15–2017/18 and 81.8% were males while the remaining 18.2% were females. Of all malaria cases treated in Abrajira Hospital during 2015/16–2017/18, 61% were PF while the remaining 38.9% were PV.

**Table 2:** Seasonal (August–November) malaria screening and treatment of laborers working in mechanized agricultural farms during 2015–2017 in mobile treatment centers and selected agricultural camps

Treatment centers	2015				2016					2017		
	Aug	Sept	Oct	Nov	Aug	Sept	Oct	Nov	Sept	Oct	Nov	Total
Mobile sites	–	–	–	–	390	–	–	–	254	1421	72	2137
G/SILASSE Camp	49	–	–	–		237	361	238	–	–	–	885
Desalegn camp	304	–	–	–	88	209	136	145	127	181	16	1206
Asmare camp	387	–	–	–	95	490	706	419	52	373	10	2532
Gosh Abraha camp	–	–	–	–	43	–	–	–	129	301	7	480
Mulalem camp	219	–	–	–	114	284	343	134	85	179	9	1367
Addisu camp	–	–	–	–	15	–	–	–	27	111	4	157
Mulugeta camp	451	–	–	–	33	282	352	214	13	276	26	1647
DrAyana camp	326	337	373	320	–	–	–	–	357	466	283	2462
Addis Hagos camp	170	–	–	–	–	–	–	–	–	–	–	170
**Total**	**1906**	**337**	**373**	**320**	**778**	**1502**	**1898**	**1150**	**1044**	**3308**	**427**	**13,043**

From the malaria cases treated in Health Posts in West Armachiho district, 6.2% were below age 5 years and 14.9% in 5–14 age group and 78.9% in above 14 years old age group. These results were similar for Quara district (8.1%) and Metema district (10%) for less than 5 years. The malaria cases and treated in 5–14 years age group in Quara and Metema were 19.5 and 17.4% respectively (Fig. 2).

Of the total malaria confirmed cases and treated in Abdurafi and Abrajira Health Clinics during 2014/15–2017/18, 6.9% (1503 males and 1565 females) was from under 5 years while the remaining 17.9% (4847 males and 3132 females) and 75.3% (26,613 male and 6977 females) were from 5 to 14 years and above 14 years respectively. Of the total above 14 years treated, 79.2% were males. Statistically significantly difference were found in under 5 years children (*p* = 0.000), 5–14 years (p = 0.000) and above 14 years age groups for male and female (p = 0.000) in the Health Clinics (Table 3).

**Table 3:** Mann Whitney U-Test to show statistically significant difference between male and female malaria confirmed and treated patients in Health posts and Health clinics

Health posts or Clinics	Age	Sex	Mean ± SD (Median) Malaria cases	Range	Total malaria confirmed	P-values	
Health Posts	≤ 4 years	M	2.3 ± 5.9 (1)	0–96.00	1503.00	P = 0.000	P = 0.000
		F	2.3 ± 21.2 (0)	0–542	1565.00		
		T	2.3 ± 15.5 (1)	0–542	3068.00		
	5–14 years	M	7.3 ± 10.2 (4)	0–66	4847.00	P = 0.000	
		F	4.7 ± 7.0 (2)	0–63	3132.00		
		T	5.97 ± 8.8 (3)	0–66	7979.00		
	≥15 years	M	39.8 ± 82.8 (12)	0–1055	26,613.00	P = 0.000	
		F	10.4 ± 19.7 (5)	0–250	6977.00		
		T	25.1 ± 61.9 (7)	0–1055	33,590.00		
	Total	M	16.4 ± 51.1 (3)	0–1055	32,963.00		
		F	5.8 ± 17.5 (2)	0–542	11,674.00		
		T	11.1 ± 38.5 (3)	0–1055	44,637.00		
Health Clinics	≤ 4 years	M	4.9 ± 8.0 (2)	0–69	929.00	P = 0.000	P = 0.000
		F	3.5 ± 5.4 (2)	0–46	664.00		
		T	4.2 ± 6.9 (2)	0–69	1593.00		
	5–14 years	M	11.2 ± 14.3 (7)	0–110	2147.00	P = 0.000	
		F	7.0 ± 9.4 (4)	0–67	1345.00		
		T	9.1 ± 12.2 (5)	0–110	3492.00		
	≥15 years	M	132.1 ± 191.1 (54)	0–1176	25,224.00	P = 0.000	
		F	22.4 ± 31.04 (12)	0–198	4282.00		
		T	77.2 ± 147.3 (22)	0–1176	29,506.00		
	Total	M	49.4 ± 125.1 (8)	0–1176	28,300.00		
		F	11 ± 20.7 (5)	0–198	6291.00		
		T	30.2 ± 91.6 (6)	0–1176	34,591.00		

**Fig. 4:**
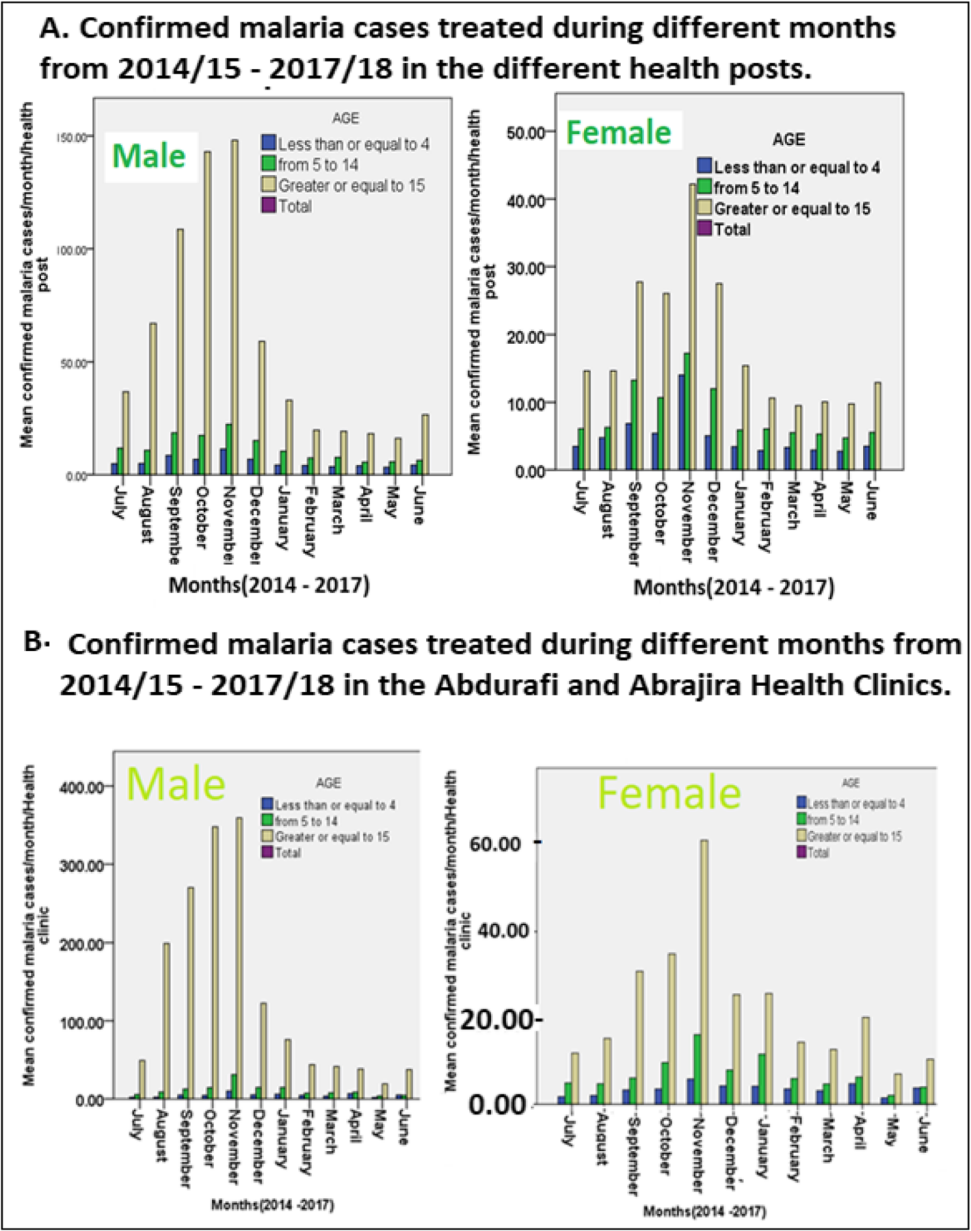
Proportion of males and females in different age groups malaria confirmed and treated in different health posts (A) and health clinics (B)

Of the total malaria confirmed cases and treated in Grar Wuha, Mogese, Durmaga, Gobla, ZemeneMerik, Torka, Enzlish and Meharish health posts, 4.5% (929 males and 664 females) were under 5 year children. The other 10.1% (2147 males and 1345 females) and 85.3% (25,224 males and 4282 females) were from 5 to 14 years and above 14 years age groups respectively. Of the above 14 years treated in the health posts, 85.5% were males (Table 2). Male above 14 years old treated 6 times higher in health clinics and 4 times higher in health posts than females. Statistically significant differences (*p* = 0.000) were found between males and females malaria confirmed and treated patients in all health posts and clinics in West Armachiho District (Fig. 4). Confirmed malaria cases were statistically significantly different in different months in Health posts and health centers in West Armachiho district where peak malaria incidences were occurred in October and November (Fig. 4).

## Discussion

Annual malaria parasite incidences in the lowland districts of West and Central Gondar Zones of Ethiopia (Table 1) during 2013 to 2017 were above 100 malaria cases/1000 population as already reported [14]. Generally, progressive reduction has been seen from 357 case/1000 people in 2013 to 130.6 cases/1000 people in 2017 in Gondar zones with the exception in 2016. In Ethiopian highlands, malaria epidemic occurs every 5 to 8 years [14]. The epidemic like 453.6 malaria cases/1000 people in 2016 in Gondar Zones might be related with normal trend of malaria epidemic associated with abnormal climate in 2016. Easy access to drug and lack of death might be the reason why such an epidemic remained unnoticed. The result of this study has showed significant reduction to 130.6 cases/1000 people in 2017 compared to 453.6 cases/1000 people in 2016. But, globally malaria cases were raised from 217 million in 2016 to 219 million in 2017 [15]. Addressing malaria transmission in mobile seasonal and permanent laborers, by putting extra-effort, most probably reduce malaria incidence significantly in different zones of Gondar and Amhara region as a whole.

During September – November 2016 and 2017 seasonal malaria screening and treatment campaign in mobile venues and some agricultural treatment centers, more than 10, 000 laborers working in the mechanized farm were treated for malaria cases (Table 2). Of all malaria treated patients in health posts and Health clinics in the West Armachiho district, males accounted for 73.8 and 81.8% respectively. When age groups of malaria patients treated in the health posts and clinics in the areas were compared, male in age groups above 14 years were accounted for 79.2% for health posts and 85.5% for health clinics. All these figures support majority of malaria cases were originated from laborers working in agricultural fields. Failure of Ethiopian Malaria Elimination Program to include these laborers and where they live during agricultural rainy season most probably increased the risk of malaria transmission both in the lowland and highland areas by re-introduction of parasites for local vectors to facilitate the transmission. In such situation, it is difficult to imagine for reaching the pre-elimination stage of the malaria elimination program. Addressing the problem of high malaria incidences in laborers working in mechanized agricultural fields in Metema – Humera lowlands is apriority.

In lowlands, June – August heavy rain months were found for high malaria transmission until peak is reached in October or November. Dry seasons without rain were also found with stable malaria transmission. In the lowland districts, weekly median malaria cases were ranged from around 100 to 600 throughout the dry seasons which must be related with the presence of the rivers near human settlements like Genda wuha, Abrajira and Abdurafi towns. Similarly, malaria transmissions in lower highlands were non-seasonal and persisted throughout the year with major peak in October and minor peak in April. Probably, the dry season malaria transmission in this lower highland malaria areas might have been related with the presence of rivers as indicated for lowland areas. March–April rain, which might have no effect on malaria transmission in lowlands, could be responsible for slight peak observed in April in lower highland areas (Fig. 3). Stable malaria transmission was also observed during dry season in upper highland malaria areas which could also been related with the presence of rivers. The dominant *Anopheles* species related with river as larvae habitat was *Anopheles cinereus* [16]. The controversial issues related to non-seasonality of malaria transmission and the presence of malaria almost in all highland areas of North and Central Gondar will be solved if the presence of *Anopheles arabiensis* (principal vector) and/or vector status of local highland *Anopheles* species is determined. Due to lack of transmission dynamics study between human and local vectors in upper highland areas including human biting behaviors, highland malaria has not been recognized officially for implementation of malaria control tools.

**Fig. 5:**
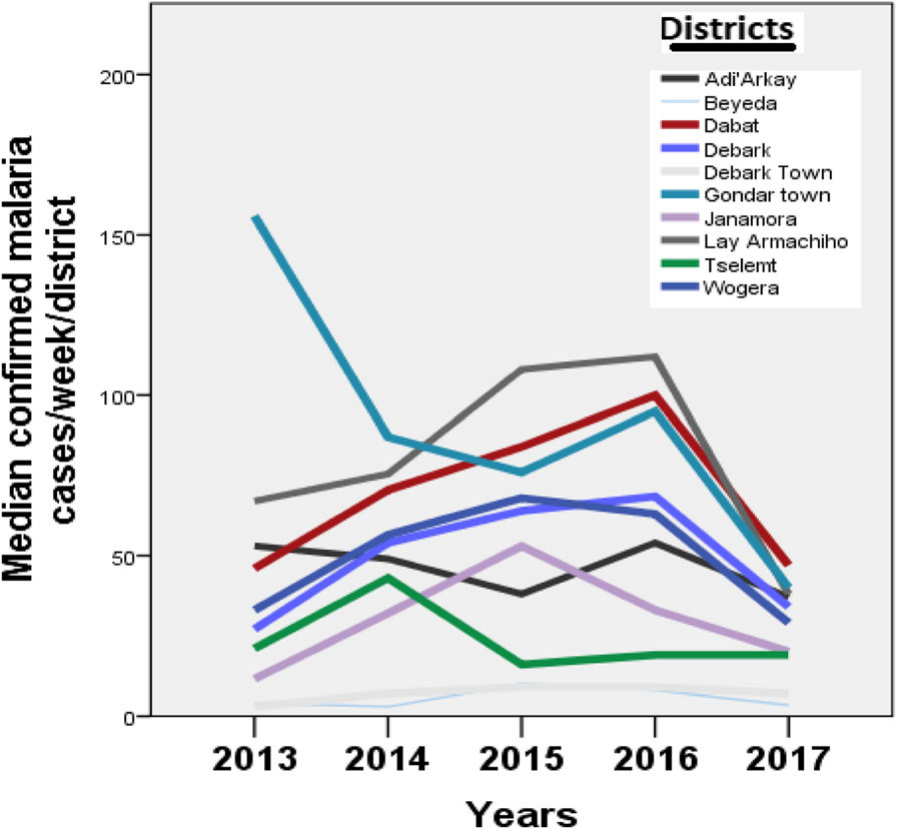
Median confirmed malaria cases/week/districts in different years in upper highland areas in Central and North Gondar zones

Kruskal-Wallis ANOVA and Median tests has indicated statistically significantly different variation in malaria cases registered during different years from 2013 to 2017 in upper highland cold areas (Fig. 5). But, fluctuation in mean or median number of malaria cases in different upper highland districts in different months or years during the study period did not show absence of reported malaria cases. Amhara Region Health Bureau is receiving continuous uninterrupted malaria case PHEM reports almost from all highland districts from North and Central Gondar.

The highest annual malaria incidences per 1000 population for Debark and Dabat towns, during 2003–2012 retrospective analysis, were reported around 25 and 50 for these towns respectively [17]. The overall annual malaria incidence per 1000 people during 2013–2017 in this study was also calculated around 24 indicating moderate transmission in the upper highland areas above 2000 m altitude. The evidence of low malaria cases in infants of under 5 years in Beyeda (0.3%) and Dabark town (0.7%) can support low transmission rate in these very cold areas. On the other hand, the highest under 5 years malaria cases compared to all North, Central and Western zones in this study was reported in Wegera highland areas (36.3%) (Fig. 2b). Such high malaria incidence in under 5 years children might be related to highly endophelic malaria vector and lack of interventions by insecticide residual spray (IRS) and distribution of insecticide impregnated bed nets. Lack of long lasting insecticide impregnated bed net (LLINs) was reported as a reason for high malaria risks in rural areas of Dabat district [18].

In all reports of Federal Ministry of Health of Ethiopia and President Malaria Initiative Operational Reports [2–5], *Anopheles arabiensis* reported as the primary malaria vector in Ethiopia and *An. funestus, An. pharoensis,* and *An. nili* as the secondary vectors. Further study may bring new vector(s) into picture that could be responsible for transmission of highland malaria especially in upper highland malarias areas since the above mentioned vectors have not been reported before.

High over all prevalence of *Plasmodium vivax*. (31.5% or 24, 039/76329) in male young and adult laborers working in agriculture fields in west Armachiho district were found during 2015 to 2017/18. High malaria incidence in agricultural laborers could play also a major role increasing and re-introducing *Plasmodium vivax* into high lands in addition to the fatal *P. falciparium* when untreated. Inability of removing *Plasmodium vivax* by treatment effectively like *Plasmodium falciparium* has already posed problem in malaria elimination program in the world [15]. Addressing the high malaria incidence of seasonal migrant and permanent laborers with appropriate control tools by including them and their work places in national malaria elimination program in Ethiopia will help the process of malaria elimination by reducing the number of *Plasmodium* parasites infections.

## Conclusion

Failure to include seasonal migrant and permanent laborers working in mechanized farms in Metema – Humera lowlands most probably have resulted in higher malaria prevalence in West, Central and North Gondar Zones. The re-introduction of *Plasmodium* parasites to the potential vectors for local transmission in highlands by returnee could be among the important problems associated with seasonal migration. To address the problem of high malaria incidence in Metema – Humera lowlands, it is necessary to design malaria control strategy that is appropriate for both upper highland areas and mechanized agricultural farms of the lowland areas.

## Data Availability

The data analyzed is available in Amhara Regional Health Buiro and the corresponding author and could be available on reasonable request.

## Abbreviations

ACT: Artemisinin-based Combined Therapies
DDT: dichloro-diphenyl-trichloroethane
IRS: Indoor Residual Spraying
LLINs: Long-Lasting Insecticidal Nets
RDT: Rapid Diagnostic Test
